# Medical Records: A Historical Narrative

**DOI:** 10.3390/biomedicines10102594

**Published:** 2022-10-17

**Authors:** Jacek Lorkowski, Mieczyslaw Pokorski

**Affiliations:** 1 Department of Orthopedics, Traumatology and Sports Medicine, Central Clinical Hospital of the Ministry of Internal Affairs and Administration, 02-507 Warsaw, Poland; 2 Institute of Health Sciences, Opole University, 45-060 Opole, Poland

**Keywords:** eHealth, healthcare system, history of medicine, medical records, patient management

## Abstract

The history of medical records is thousand-year-long, with earlier roots in ancient civilizations. Until the 19th century, medical records mainly served educational purposes, later assuming other roles such as in insurance or legal procedures. This article comprehensively describes and reviews the development of medical records from ancient to modern times in Europe and North America, reflecting alterations and adaptations compliant with the mental and technological capabilities of a given period. We searched PubMed and Google Scholar databases to collect pertinent articles. English articles or those having English abstracts were considered. The search terms included “Medical Records,” “Health Records,” “History of Medicine,” and “eHealth” and covered the last hundred years. References were also picked out from the identified articles. Overall, 600 articles were identified, 158 of which were judged thematically relevant. The general conclusion is that medical records undergo a revolutionary change from paper-based to electronic format, which reflects the development of eHealth systems. The migration process to eHealth records involves the use of artificial intelligence (AI) algorithms that streamline medical services by using faster and simpler working methods. AI benefits both patients and providers as it improves patient management and communication among medical centers, spares resources, identifies contamination or infections, and limits health costs. These advantages have become pointedly apparent during the recent COVID-19 scourge.

## 1. Ancient Times and Middle Ages

**Figure 1:**
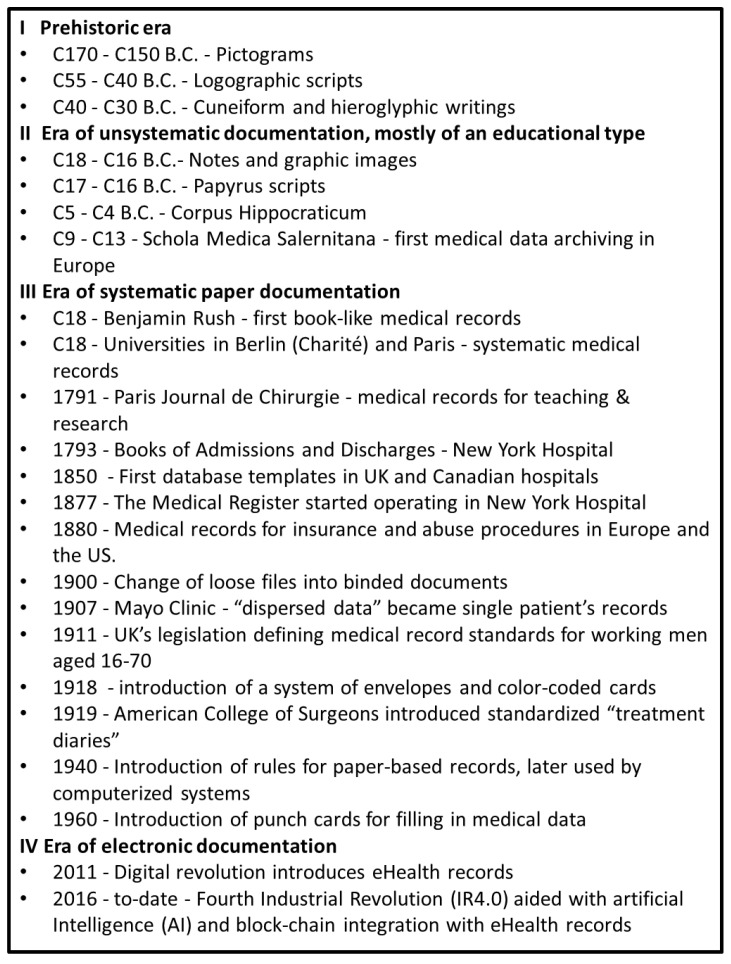
An overview of the history of medical records; C stands for ‘century.’

Human history shows that a prominent feature of Homo sapiens is intentionally leaving behind traces of one’s actions, shown by cave paintings created 17,000–15,000 years ago in the early Magdalenian period, unearthed in the Lascaux cave complex in the Nouvelle-Aquitaine region of southwestern France. One of the paintings shows an injury of a man attacked by an animal. It is arguably a pictogram of the first available medical record illustrating a probable multi-organ injury. Along with the development of the first civilizations, writing became a tool of communication, enabling the recording of a person’s knowledge. Proto writing appeared for the first time in the Vinča culture (5500–4000 years ago), while a more advanced logographic script was mastered by Egyptians, Elamites, and Sumerians circa 4000–3000 B.C. The first informed cuneiform writing consisting of hieroglyphics is believed to originate in Egypt and Sumer of southern Mesopotamia circa 3000 B.C. [1,2,3,4,5,6,7]. The first systems of noting down words and pictures were promptly picked up for recording disease-related events at the dawn of recognition of the health concept (Figure 1).

Medical records, as defined for this narrative, identify the patient and document, in written and graphic forms, all detail about his health history, clinical symptoms and signs, diagnostic and treatment procedures, medications and justification for their use, and the follow-up continuity. The research conducted by historians, archeologists, and physicians failed to establish the exact time when medical records first appeared in the ancient world. Nonetheless, it showed the significance of recording the history of illness for shaping medical knowledge over time [8,9,10,11,12,13,14,15]. Medical records, similar in structure to modern ones, were ancient Egyptian medical papyruses developed for educational purposes. In 1862, Edwin Smith, an American Egyptologist, acquired a papyrus manuscript written between 1600–1700 B.C., which is the oldest medical script about injuries. It describes the methods of examination and the determination of diagnosis, ending up with a treatment plan. Another example is “Papyrus Ebers,” acquired by Georg Ebers, a German Egyptologist and novelist, in 1873. The script, dating back to circa 1550 B.C. and now considered lost, was for millennia an extensive source of knowledge on treatments, surgical procedures, and healing herbs [16,17].

Modern medicine has been greatly influenced by the Hippocrates of Kos (460–370 B.C.). Treatment plans, ethical rules, and laws worked out in his school of thought and described in the book Corpus Hippocraticum formed the foundation of medicine. The book, a compilation of about 70 medical scripts, was written by Hippocrates and likely other physicians of the time in the second half of the 5th century and the first part of the 4th century B.C. The script structure resembles that of modern medical records, including physicians’ recommendations, descriptions of medical procedures, and prescriptions. Elements of medical law are also included, but they are mainly brought up for academic purposes. Concerning traumatology, the Corpus Hippocraticum mentions neuro-orthopedics with recommendations regarding the treatment of scoliosis and often holistic clinical observations about prosthetics, podology, and bone fracture treatment. Plausibly, knowledge encapsulated in this milestone work is much older than the script itself, but its sources are untraceable, being destroyed, like most others of the Hellenic times, in the fires of the Library of Alexandria that was established by Ptolemy I Soter in 283 BC. Europe learned about the content of the Corpus Hippocraticum no sooner than in 1525 when it was translated and printed in Venice. However, a full enactment of Hellenic medical records is impossible since only some of the scripts in the Corpus Hippocraticum meet the criteria of “records” in the contemporary sense [18,19,20,21,22,23,24,25,26,27].

The rules and knowledge passed on by Hippocrates were introduced to the Roman world by Claudius Galenus (130–200 AD), known as Galen of Pergamon, a Greek physician, surgeon, and philosopher. Galen symbolized the achievements of Roman medical culture in the centuries to follow. Like Hippocrates, he created educational health records. He was an accomplished physician whose influence continued into the 17th century. The downside was, however, that this influence did not necessarily speed up medical progress as Galen complied with the Roman law banning post-mortem examinations as of 150 B.C. The law adversely affected the development of medical knowledge and the way they were registered, even though it laid the foundation for the modern European legal system [28,29,30,31].

Medical knowledge heavily drew also from scripts created by the Islamic civilization of the early Middle Ages. There were two influential figures of that culture: Abū Bakr Muhammad ibn Zakariyyā al-Rāzī, known as Rhazes (865–925 AD), and Abū Ali Husain ibn Abdallah Ebn-e Sina, known as Ibn Sina or Avicenna (980–1037 AD). Rhazes passed on a compilation of Arabic achievements in medical knowledge of the time underpinned by past advances made in the entire Hellenic world. He also drew knowledge from Indian settlements having roots in the earliest well-organized human civilizations of Harappa and Mohenjo-Daro, which existed around the Indus River. Assumedly, those people were the first to introduce observation and inspection, a time-proven canon of medical practice. The most famous work by Rhazes was the nine-volume “Al-Kitab al-Hawi” (The Comprehensive Book on Medicine or Continens Liber in Latin). It contained clues for and elements of creating what we nowadays define as medical records and may be considered a fundamental cast for shaping such records [32,33,34,35,36]. Ibn Sina, on the other hand, was erudite and a polymath fluent in reading the Qur’an at the age of 10. He studied law and natural sciences, which helped him develop an analytical approach in his medical texts encompassing over 400 books. The most fundamental of which was “Kanun fi’t-tibb” (The Canon of Medicine), in which medical knowledge was well organized. The edition consisted of five books, each of which was divided into parts and chapters describing a variety of diseases based on past medical records. This encyclopedic edition, resembling the structure of common law, became a tenet of medical education way beyond the 18th century [37,38,39,40,41,42].

Moses ben Maimon (1138–1204), known as Maimonides, was a celebrated rabbi, philosopher, and physician. The complex political and religious situation in Spain made him leave Europe and settle in North Africa. He became a doctor to the vizier Al-Fadhil, a regent of Egypt. In his medical practice, he noticed a connection between the “psyche” and “soma,” which was strongly influenced by his philosophical and religious background. His guiding principle was to “treat the ill, not the illness,” and his most famous work, “Aphorisms of Moses (Pirkei Mosche), presented clinical descriptions of many a disease. He encouraged preventive healthcare and was convinced that the key to a healthy life was in one’s relationship with nature, surroundings, and moral values. He raised the idealistic concept of a “doctor-patient” interaction we wishfully expect today. His scripts can be taken as educational medical records [43,44,45].

The word ‘hospital’ springs to mind when discussing medicine and medical records. In medieval Europe, unlike today, hospitals were treated as asylums for the poor and ill. They were managed mainly by convents, which was an effect of the Christian moral imperative to do good and show mercy to the needy. There were exceptions to this rule as some institutions were improving disease knowledge and management, which was reflected in medical records of the time. A case in point was the Schola Medica Salernitana, famous in medieval Europe but often forgotten today. Steady progress was also made at the level of individual cultures and civilizations [46,47,48,49]. Civilizations of the time were functioning independently from one another, so medical records differed accordingly. The primary purpose of records was determined by administrative organs, almost always connected to the Church. The lists of patients admitted and released from hospitals were kept in religious institutions and are today considered the first examples of medical data archiving in Europe. Medieval medical records can be considered more autonomous than ancient ones, and the habit of documenting medical procedures or observations became a constant element of doctors’ practice. Medical records throughout history had a narrative character, changing somewhat depending on the period in which they were written. The facts argue that those from ancient times were intellectually much above their medieval counterparts [4].

## 2. Modern Times

Changes in the approach to medical records came with the renaissance and the work of Leonardo da Vinci (1452–1519). Research performed by just one person appeared as a stepping stone in many sciences, including medicine. The development of numerous branches of medicine would have been difficult to imagine without da Vinci’s anatomical sketches. Handmade sketching became a way of producing educational records used, particularly in orthopedics and the like, until the end of the 20th century. Currently, however, such sketches do not meet the requirements of eHealth records anymore [50,51]. Another figure who greatly influenced the way medicine was run was Andreas Vesalius (1514–1564). In 1534, he published “De Humani Corporis Fabrica” (On the Fabric of the Human Body), which revolutionized the field of medicine. Medical records began to include post-mortem sketches. Those dedicated to Vesalius’ book were made by Jan van Calcar, a pupil of Titian. Rembrandt Harmenszoon van Rijn (1606–1669) depicted Vesalius’ book on canvas in the right lower corner of the famous medical painting “The Anatomy Lesson of Dr. Nicolaes Tulp” [52,53,54,55,56,57].

It is difficult to establish firm connections between renaissance science and the arts. The intellectual elite of the time consisted of few people. Yet universities communicated and collaborated. The process of promoting anatomical knowledge included planning and conducting post-mortem examinations, which allowed the unraveling of about 200 inconsistencies in the Galen publications, considered exemplary at the time. New knowledge enabled the resketching and rewriting of results, leading to enlightening feedback discussions. As a result, the relevance of Galen’s view of medicine started fading away, irrespective of whether discoveries were intentionally planned or serendipitous.

The 17th century brought a rapid development of natural sciences in Europe caused by renaissance-driven curiosity. Post-mortem examinations were conducted on a scale unheard of before, which provided material for a gigantic number of medical records. The phenomenon stimulated the development of science. While discussing medical records created during that time, the case of Philip Verheyen (1648–1710) should be necessarily recalled. He had his left leg amputated during the second year of his studies. As he was deeply religious and wanted his body to be buried intact, awaiting resurrection, he kept his amputated limb in a substance preventing decay. In 1693, he performed post-mortem examinations on the amputated leg, which resulted in the discovery of the Achilles tendon. Despite personal tragedy, he further contributed to medical science, giving the first description of phantom pain in a professional and skilled manner, which still serves as an archetype example today. As the concept of phantom pain was unknown in the 17th century, curiosity made him begin studying anatomy. His notes compiled between 1700 and 1710 were published as “Letters to My Amputated Leg” [58,59]. He also wrote the book “Corporis Humani Anatomia,” which was considered the best medical textbook by most European universities at the beginning of the 18th century [60,61]. Nonetheless, the interest faded away due to imperfections and unclarities found in his descriptions as science progressed with time.

The number of medical records in the form of sketches and descriptions made until the beginning of the 18th century is difficult to estimate. Europe lost many a book being torn by the Thirty Years’ War and the Great Northern War. At least half of parish registers, considered the most important documents at that time, were destroyed. Physicians of Western Europe were unlikely to run medical records diligently in the mid-18th century, and only a fraction of the records unraveled have been researched [62,63]. An exception is an accomplished American physician, Benjamin Rush (1745–1813), educated in Edinburgh, who kept detailed medical records of patients in the form of a book. Nowadays, his work is considered the archetype of medical history [4]. The character of hospitals began to change at the end of the 18th century. The hospital was no longer seen as an asylum for the poor but rather considered a medical center. Changes also affected the doctor-patient relationship. This time marks the beginning of the modern medical record system written in national languages like German rather than Latin [64]. Historians point to another event as a stepping stone in the process of formalizing medical records. At the beginning of the 18th century, military surgical courses were moved to universities in Berlin and Paris to be later transformed into medical schools, which developed their procedures and methodologies, including those regarding medical records [65,66,67].

In 1724 in Berlin, the capital of Prussia at the time, a garrison hospital was rearranged into the surgical collegium, later named Charité by Frederick William I. The first head of the institution was Johann Theodor Eller (1689–1760), a Royal Doctor. A collegium’s routine was the everyday inspection of patients conducted by junior surgeons, which involved writing up the patient’s condition and the history of treatment in the form of a journal. Johann Theodor Eller considered it the best form of education, which enabled the achievement of new skills by doctors and benefited patients. He introduced a hierarchical system in which medical records formed a way of communication between experienced physicians and their younger underlings. Such modern ideas fit well into the concept of enlightened absolutism, a Prussian version of Enlightenment. The strong centralized political power of the monarch supported by the developing bureaucracy, which also influenced the way of running medical records, became an example to follow in institutions like the Charité—Berlin University of Medicine, Europe’s largest university hospital [68,69,70].

The Hôtel-Dieu hospital in Paris became a center for the development of the 18th century’s medical education thanks to Pierre Foubert (1696–1766) and Pierre-Joseph Desault (1744–1795). Patients had obligatory daily check-ups that provided data needed for research. In 1791, Pierre-Joseph Desault established the Journal de Chirurgie, in which he described and commented on the most interesting cases he had come across [69,71]. For the first time in modern Europe, the concept of in-depth medical records became not only a set of tips for treating patients but also a base for medical research. At that time, a uniform way of registering patients was not yet in place, but advancements were made, with Paris and Berlin taking the lead.

## 3. Recent Centuries

The US started a system of patient case records independently from Europe. The cornerstone was the introduction of the Book of Admissions and the Book of Discharges in the New York Hospital in 1793. The Governor Council of the State of New York set a medical register and hospital rules at that time as well [72]. About a decade later, doctors David Hosack and Alexander Hamilton proposed that home doctors should register all medical cases. The aim was to preserve the knowledge in a written form, which could later be used by medical students. The proposition was implemented by the Council [73,74]. Unfortunately, entries in the register were initially few and retrospective rather than taken at the bedside, and some were personal notes suggesting that moral behavior toward patients was often misunderstood [75]. Restrained bureaucracy at the time enabled physicians to display a sense of their style. The length of entries varied depending on the complexity of medical problems and the physicians’ approach to them [76]. The structure also varied depending on the physicians’ creativity and mood, which is shown in the exemplary fragments of an entry, quote/unquote “Now it is a partial paralysis of both touch and movement, of both upper and lower limbs, he cannot walk … without a stick … What troubles him now are overgrown testicles … He said he had night sweats … He does not have rheumatism or syphilis and says he has no appetite, but he was practicing masturbation. He lost a lot of energy, is pale, and his left side is worse; his mind and eyesight are alright, and he is a hypochondriac …” Despite the efforts of the Governor Council, which hired so-called conservators to supervise the registers, the entries were far from acceptable considering modern standards. Textbook structures were not much more professional, as even making derisive fun of patients was considered acceptable [73]. Hospital boards of the 19th century established rules for creating medical registers according to what nowadays could be called a vision of the organization [76]. Then, records often reflected cultural stereotypes, personal medical theories, and philosophies of their authors. It soon became apparent that it was insufficient or wrong, and register entries must be up to certain standards [75]. The necessity also appeared to create a database of acronyms and abbreviations to streamline meaningful communication among medical professionals. Because the Governor Council of the State of New York required annual reports, staff duties regarding medical records were defined, including hospital admissions, discharges, treatment results, and expenditures, which enabled the documentation of achievements. It followed that as of 1830, the number of patients taken care of was linked to the prospect of the doctor’s promotions [73].

Similar changes concerning medical records were also happening in Europe. Historiographical investigation shows that some medical records of the 19th century resemble the ones of today [12,77]. Up to the beginning of the 19th century, diagnostics in Europe and the US were based predominantly on anamnesis, i.e., interviewing a patient, while the physical examination was less crucial. The change in practice came around with Dominique-Jean Larrey (1766–1842), a physician to Emperor Napoleon I, who was a surgeon in Val-de-Grâce Hospital in Paris and a pioneer of combat surgery. He considered the examination of a patient the priority. Meanwhile, a German view on laboratory medicine increasingly pointed to the need for recording and analyzing pure data, shaping the way medical records were conducted [58,78]. The registries of patients, which began in Paris and Berlin, made way for statistical elaborations, creating the foundations for epidemiology, clinical research, and evidence-based medicine [4,12,79]. Barbara L. Craig investigated the 1850–1950 databases of four hospitals in London and Ontario (Canada) and concluded that this historical period was crucial for the commencement of a modern medical record system [80,81]. Changes became noticeable in the mid-19th century when doctors started registering data of all patients. Universal templates of records were introduced to avoid confusion during presentations of medical cases at conferences [73,78,82].

Growing specialization in health care, which emerged in the second half of the 19th century, affected the structures of hospitals and the form of medical records. The sheer number of records became steadily larger. They were also copied and cultivated in libraries; the procedure was implemented for research and educational purposes in 1908 [72,75,82,83]. Classic examples of a full history of an illness in the Anglosphere include a referral letter for examination from a doctor, suggesting diagnostic procedures, epicrisis, an analytical evaluation of the case management and treatment, and the continuity of care, and casuistry, the resolving of clinical doubts or issues based on knowledge and reasoning. At the turn of the 20th century, the technique of administration of reports changed from loose segregated files to binding documents and collecting them into folders after patient discharge. Stamping documents stamp was originally needed for bookkeeping, confirmation of the patient’s registering, and formalizing medical records, which was called by some authors the introduction of business techniques [80,81,84]. The authors of reports also had to list their names in chronological order [85]. In 1898 in the US, records created at the bedside were considered complete medical records in today’s sense rather than just notes for educational purposes. Walter Bradford Cannon (1871–1945) pioneered the use of medical records for teaching at Harvard Medical School [4,78]. Nonetheless, the content of records was still limited as they included a family interview, eating habits, drugs used, prior diseases, current disease, results of physical examinations, investigations of blood and urine samples, treatment regimen, and diagnosis. Patients’ data were often dispersed between wards and ambulatories, so finding the details needed was difficult and often depended on the memory of the record’s author. The situation was similar in both public and private practices [83,86].

Investigating medical archives from 1810–1932 in the New York Hospital, it can be noticed that the number of records was steadily rising, as pointed out by Ryann L. Engle during his research on palsy and atony [72,73]. At the turn of the 19th/20th centuries, medical records in the US and Europe structurally began resembling the present ones. Information about an individual patient could be retrieved just by using his data. Nonetheless, the recording and retrieval systems kept evolving toward improvements. The Rockefeller Foundation considered medical records a crucial element in enhancing the quality of health care and education, as pointed out in the Abraham Flexner Report of 1910 [87]. Henry S. Plummer (1874–1937) was the first who solved the issue of “dispersed data” by applying a single record to each patient, just the way it had been conducted in business and industry. This essential change took place at St. Mary’s Hospital and Mayo Clinic in 1907. Yet medical records remained incomplete compared to the present ones due to the lack of epicrisis [4,88].

In the 1860s, handwritten diagrams of life parameters, fever cards, pulse and breath measurements, and urine diagrams became common. Their form was up to physicians. Diagrams were also used to assess the dose of morphine in the treatment of peritonitis. The Medical Register became fully operational when a building intended for this purpose was opened in the New York Hospital. Since 1877, all medical records have been stored and supervised there [73]. Introducing a universal history of the disease, diagrams, and forms became common practice at the beginning of the 20th century [86,87]. Stanley Reiser described the introduction of such arrangements in the Massachusetts General Hospital [89,90,91]. The way of making medical records followed the models already used in economics. Such models, displaying information in a graphic form, proved effectively manageable.

Since 1880, medical records in the US and Europe became relevant to insurance or abuse procedures. In the UK, a law was enacted in 1911 defining the standards of how to keep medical records concerning social insurance mandatory for working men between 16 and 70 years of age. A system of envelopes and color-coded cards was introduced and used until the 1970s [4,73,88,92]. It is impossible to establish, based on the resources available, whether Europe was the first continent where medical records were firmly established for use. However, a simple transfer of American procedures to the Old Continent seems unlikely due to the cultural dominance of Europe over the US before the First World War. The ultimate answer to this question requires more research.

In 1916, written recommendations appeared in the US to put down basic information about diseases in a standardized form, which may be considered predecessors of modern ICD 10. In 1918, the American College of Surgery decided to register all patients in all hospitals to better monitor the treatment and compare the results if necessary. The measure was not entirely effective as medical records were often illegible, which constrained its advantage [93,94]. Nonetheless, the importance of medical records was apparent. Joel D. Howell, who described the modern hospital as an institution, considered medical records a part of modern medical technology. He noted that the development of specializations produced professionalized health records, which was necessitated by an increasing number of diagnostic and therapeutic procedures and documentation engaging more employees [92]. An opinion circulated among American surgeons, the first physicians who implemented the gathering of health records, that affiliation with academic environments provides superior qualifications. In 1919, the American College of Surgeons began a standardization campaign using the “treatment diaries.” A diary must include an interview with the physician, all laboratory tests, diagnosis, the chronology of a treatment plan, and daily discourse. Importantly, data were archived. Many hospitals supported the initiative. Offices and administrative networks were set up to keep the centralized registers in order. Hospitals started hiring professionals to handle data derived from records. According to Stefan Timmermans and Marc Berg, these processes began in the US, thanks to the activity of the medical community and then spread to Europe [79].

In the 20th century, a new problem arose, which is the presence of spam files, i.e., unwanted and unsolicited messages which create chaos in clinical reports and observations. It became clear that quality checks were needed. The concern about the uncontrolled changes applied to medical records has been expressed for nearly 100 years now [78,95,96,97,98]. Advancements in the paper-based systems of medical records were brought on by the Second World War [4]. Some of the algorithms then used to organize the records are still used by modern computerized systems.

## 4. Digital Revolution—eHealth Records

Drastic changes consisting of the introduction of eHealth records began in the 1960s. Initially, data were filled in using punch cards, which was a tedious process. However, it enabled the collection of data from diagnostic procedures for later evaluation and use in research, education, therapy, economics, and administration in a much more efficient way than in the case of paper-based documentation [88,92,99,100,101]. An investigation of medical institutions in the US showed that only 10% of them had a large-scale computer system before 2009. Medical records were still paper-based. Afterward, the Health Information Technology for Economic and Clinical Health (HITECH) recommended that medical centers must introduce eHealth record systems [89]. In 2011, nearly 50% of US physicians used eHealth record systems, thanks to streamlining the software and decreasing costs, although doubts about eHealth efficacy persisted [90,91,102,103]. Currently, about 80% of hospitals and physicians’ offices use eHealth systems comprising huge databases of information for treatment plans, clearance of medical procedures, research, and costs. A feedback mechanism matches the functioning databases with search and analysis programs. Increasingly, those programs make use of advances in modern information technology and artificial intelligence; and vice versa, artificial intelligence has been shown to inform the creation of new databases. Databases of eHealth records fulfill a multitude of functions. They propagate the practice of evidence-based medicine, assist with diagnosis and treatment, help predict pandemic outbreaks and reduce medical errors [104]. Despite obvious advantages, 20% of respondents representing various types of medical professionals still express doubts about benefits stemming from eHealth records [4,88,105]. The arguments raised involve potential inadequacies such as disagreement with current requirements, unfriendly interface, extensive costs, and a lower standard compared to that used for business [91,103].

In Europe, currently, eHealth records are common but are supported by paper-based elements to a different degree in various countries. A flagship example of a complete eHealth record system is that introduced in Estonia. Digital records of health information include the patient’s demographics, health history, doctor visits, treatment courses, medications, procedures, immunizations, hospitalizations, lab tests, and other pertinent administrative and clinical data. Over 150 Estonian organizations use the X-Road network, a database for digitalized documents, with all hospitals being connected to it. The Estonian National Health Information System has been created to replace scattered paper-based sources. To access the eHealth system, one needs an ID card that is physically inserted into a computer. The system is based on blockchain technology based on the peer-to-peer network that makes the files decentralized and usable by multiple people at the same time. This technology stores and distributes information that is then encrypted by algorithms called cryptographic hash functions, which, aside from medical records, are used in online transactions or bookkeeping. Estonian society is practically free of paper-based documents, which helps save 2% of GDP annually. There are just three formal matters that cannot be taken care of online: marriage, divorce, and inheritance [102,103,104,105,106]. Similar eHealth records also are fully operational in Scandinavian countries and the Netherlands as of 2019. A centralized system of eHealth records at the national level is the most cost-effective and offers the benefit of a holistic view of a patient’s history, which is accessible by the patient as well as different healthcare providers and authorized professionals. Nonetheless, the integration of a national healthcare system raises several organizational and fiscal challenges linked to the ways of sharing communication and information, fiscal participation of healthcare providers, processing of multiple not always consistent datasets, and other hurdles, which may delay the system’s interoperability at the national level [107].

Considering the current COVID-19 pandemic, the introduction of eHealth records could be beneficial considering coordination among hospitals. Symptoms of COVID-19 are hardly different from those of the flu, so finding common patterns among larger numbers of patients would improve the diagnostics where specialized tests are inaccessible or inconclusive. The full extent of the pandemic is yet unsettled. Elizabeth Halloran, a biostatistician at the Fred Hutchinson Cancer Research Center in Seattle, Washington, estimates the real number of infected people in the US at 5–20 times higher than the numbers given. Italy’s Civil Protection Agency suggests a ratio of 1:10 confirmed infections. With the numbers of infected people varying this much, it is difficult to perform any epidemiologic modeling. eHealth records of the histories of infections could help estimate the true number of infections in a society. Another advantage would be that knowing the patient’s past or coexisting diseases might be essential for the assessment of the COVID-19 threat for post-infection complications or death. Limiting the amount of paper-based documentation, often prepared next to the patient and then passed along to the medical staff, would also minimize the surface-contact route of contamination with the virus that causes disease [108,109].

## 5. Conclusions

The present view on medical records reflects the progressive alterations and adaptations of procedures compliant with the mental and technological capabilities of a given period. Future developments are posed to vary substantially from today’s [110,111,112,113,114,115]. Drawing time-proven experience, the traits we value in medical records nowadays have well-organized structure and clarity. Medical records lacked formalization for millennia, and the modern systematic approach is an achievement of the last 100–150 years. Another crucial aspect is the records’ quality. Often, a medical script that took a significant amount of time to create was later considered useless due to a lack of readability. Many an author says that the quality of medical records cannot be judged when the described cases and patients are anonymous. Records were not meant to last for centuries, although the value of some of them has withstood the test of time. The records’ worth might have been influenced by promoters of specific treatment strategies. Another factor to be considered is the “magical power” of a big cluster of data, which, even when of dubious quality or value, might incidentally or sometimes delinquently create an aura of professionalism. The capability of selecting and analyzing data from historical records is crucial [116,117]. The recorded history of disease is considered a valuable educational tool, providing that the data and descriptions are well-balanced and complement each other. The comprehensiveness of an overwhelming volume of medical records requires their structure and writing style to be adapted in line with advancing technology. Yet the disease history still rests on personal notes and epicrisis written by a physician. In some healthcare systems, the epicrisis is replaced by a letter to a general physician [118]. Medical records are not to be mistaken for the history of medicine. While the records comprise original documents and observations made by physicians, the history of medicine is often written by a single person in the form of a closed chronicle. In contrast, the patient’s history is compiled as a single folder or file. When the process is digitalized, only the last saved version of the file is legally binding [4,12]. The ongoing transformation of paper-based records into eHealth solutions makes medical services more manageable and flexible, health prevention-oriented, and cost-effective, all to the benefit of patients. The present narrative on medical records did not tackle their history in the cultures of the Far East. Clinical and academic achievements of Chinese and Japanese medicine are recognized worldwide, which may be exemplified by acupuncture, herbal, or rehabilitation medicine [119,120,121]. The achievements took root in ancient traditional medicine and were also based on progressive medical records. The pre-modern Western and Eastern pathways of medical records were largely separate due to language-related constraints in the information flow in the bygone times. Their mutual interaction is a little-known issue that calls for a separate in-depth exploration. However, recent worldwide digitalization based on artificial intelligence blurs regional differences in shaping eHealth records.

## Data Availability

Not applicable.
